# Irisin and Metastatic Melanoma: Selective Anti-Invasiveness Activity in BRAF Wild-Type Cells

**DOI:** 10.3390/ijms26020652

**Published:** 2025-01-14

**Authors:** Simona Serratì, Roberta Zerlotin, Michele Manganelli, Roberta Di Fonte, Manuela Dicarlo, Angela Oranger, Graziana Colaianni, Letizia Porcelli, Amalia Azzariti, Stefania Guida, Maria Grano, Silvia Concetta Colucci, Gabriella Guida

**Affiliations:** 1 IRCCS Istituto Tumori Giovanni Paolo II, 70124 Bari, Italy; s.serrati@oncologico.bari.it (S.S.); r.difonte@oncologico.bari.it (R.D.F.); l.porcelli@oncologico.bari.it (L.P.); a.azzariti@oncologico.bari.it (A.A.); 2 Department of Precision and Regenerative Medicine and Ionian Area, University of Bari, 70124 Bari, Italy; roberta.zerlotin@uniba.it (R.Z.); manuela.dicarlo@uniba.it (M.D.); angela.oranger@uniba.it (A.O.); graziana.colaianni@uniba.it (G.C.); maria.grano@uniba.it (M.G.); 3 Department of Translational Biomedicine and Neuroscience, University of Bari, 70124 Bari, Italy; m.manganelli1991@gmail.com (M.M.); silviaconcetta.colucci@uniba.it (S.C.C.); 4 School of Medicine, Vita-Salute San Raffaele University, 20132 Milan, Italy; drstefaniaguida@gmail.com; 5 Dermatology Clinic, IRCCS San Raffaele Hospital, 20132 Milan, Italy

**Keywords:** myokine, irisin, melanoma, BRAF, MMPs, fibrinolytic system

## Abstract

Irisin is a newly discovered 12 kDa messenger protein involved in energy metabolism. Irisin affects signaling pathways in several types of cancer; however, the role of irisin in metastatic melanoma (MM) has not been described yet. We explored the biological effects of irisin in in vitro models of MM cells (HBL^wt/wt^, LND1^wt/wt^, Hmel1^V600K/wt^ and M3^V600E/V600E^) capable of the oncogenic activation of BRAF. We treated MM cells with different concentrations of r-irisin (10 nM, 25 nM, 50 nM, 100 nM) for 24 h–48 h. An MTT assay highlighted that r-irisin did not affect the proliferation of MM cells. We subsequently treated MM cells with 10 nM r-irisin, corresponding to the dose exhibiting biological activity in vitro. Irisin reduced the invasive ability of only LND1^wt/wt^ (*p* < 0.05), which highly expressed αv gene levels, but did not affect the invasion of BRAF^mut^ cells. Gelatin zymography analysis showed a reduction in the enzymatic activity of MMP-2 and MMP-9 in BRAF^wt/wt^ cells treated with 10 nM r-irisin. Moreover, gene expression analysis (qPCR) of *MMP-2* and *MMP-9* and of the fibrinolytic system (*uPAR*, *uPA* and *PAI-1*) highlighted a crucial role of 10 nM r-irisin treatment in the inhibition of pro-invasive systems in BRAF^wt/wt^. In conclusion, our results may suggest a possible differential role of irisin in melanoma cells.

## 1. Introduction

Irisin is a 12 kDa messenger protein that is part of the fibronectin type III domain containing 5 (FNDC5) protein mainly secreted by skeletal muscles upon muscle contraction [1]. Recently, the integrin αV/β5 has been identified as the receptor for irisin on osteocytes [2,3,4]. Through autocrine, paracrine, and endocrine signals [5] involving the activation of ERK cascade [6,7], irisin also exerts pleiotropic anabolic effects in several tissues, including the brain [8,9], heart [10] and liver [11].

It has also been proven that its has modulation effects on cellular proliferation in different cancers [12,13,14,15,16,17,18,19]. Furthermore, hepatocellular carcinoma cells treated with irisin displayed a more aggressive phenotype [20]. These findings were largely obtained from in vitro models of solid tumors, even though the effect of irisin on metastatic melanoma, one of the most aggressive forms of the tumor, has not been described yet.

Melanoma is an aggressive type of tumor that mainly occurs on the skin, with poor prognosis for patients with metastatic disease. The genetic determinants of melanoma occurrence are largely known, highlighting the pivotal role of BRAF serine/threonine-protein kinase as a driver proto-oncogene for the target therapy [21,22,23]. About 50% of melanomas are characterized by the presence of BRAF-activating mutations. As for V600E, oncogenic activation of V600K-BRAF is associated with the over-expression of the ERK pathway, which in turn leads to the over-expression of several genes involved in tumor development. Different studies on molecular mechanisms underlying metastatic melanoma progression highlighted the central role of biochemical pathways involved in endoplasmic reticulum stress, autophagy, and translational reprogramming [24,25,26]. Moreover, several proteins with proteolytic activity orchestrate several interactions between melanoma cells and the surrounding ECM tumor microenvironment. Accordingly, cell–ECM interactions regulate molecular processes which underlie cell differentiation [27], cell homeostasis [28], wound healing [29], and cancer cell invasion and metastasis [27,30,31]. Different proteolytic enzyme systems, including the urokinase-type plasminogen activator (uPA) and the MMPs family, contribute to tumor progression and therefore drive therapy decisions [32]. The uPA and its inhibitor, plasminogen activator inhibitor 1 (PAI-1), are proteins of the plasminogen activator system which proteolyzes ECM components. Elevated expression of uPA and its receptor (uPAR) are associated with poor prognosis and metastasis occurrence [33]. Plasmin further promotes the activation of matrix metalloproteinases (MMPs) either directly or indirectly [34]. In particular, the balance between MMPs and their tissue inhibitors (TIMPs) is also critical in determining tumor cell invasion and therefore melanoma tumor progression and metastasis [35,36].

Therefore, the main aim of the present work was to explore the direct effect of exogenous recombinant irisin (from here on r-irisin) treatment on proliferation and invasion in in vitro models of metastatic melanoma (MM) cell lines to further elucidate the role played by irisin in melanoma cells too.

## 2. Results

### 2.1. Effect of Irisin on Metastatic Melanoma Cell Viability

**Figure 1:**
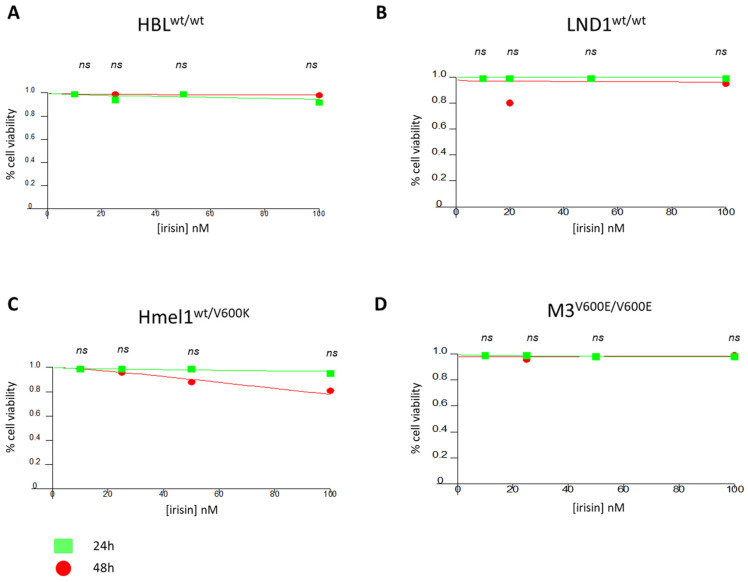
MTT-assay. Metastatic melanoma cell lines were treated with different concentrations of r-irisin (10 nM, 20 nM, 50 nM, 100 nM) for 24 h and 48 h (**A**–**D**). MTT assays were performed with at least three replicates per experimental condition. ns = not significant.

In order to explore the effect of irisin on metastatic melanoma (MM) cell viability, we treated the MM cell lines HBL^wt/wt^, LND1^wt/wt^, Hmel1^V600K/wt^ and M3^V600E/V600E^, characterized by the oncogenic activation of BRAF, with different concentrations of r-irisin, for 24 h and 48 h. As show in Figure 1, based on the concentrations (0 nM, 10 nM, 20 nM, 50 nM, 100 nM) reported in the literature [3,12,13,14,15,16,17,18,19], irisin did not impair the viability of MM cells at 24 h and 48 h after treatment compared to untreated cells (ns = not significant).

### 2.2. Irisin Reduced Invasion in LND1^wt/wt^ Melanoma Cells

**Figure 2:**
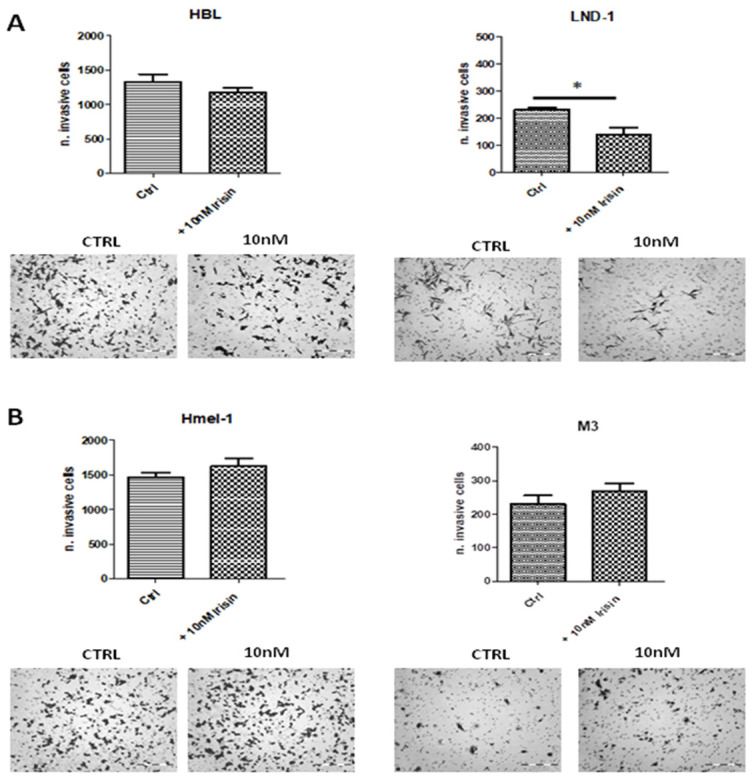
Chemio-Invasion assay. Melanoma cells were migrated through Matrigel-coated porous filters in a 10 nM irisin gradient, showing a reduction in the invasion potential of (**A**) HBL^wt/wt^ and LND1^wt/wt^ compared to untreated (* *p* < 0.05) and (**B**) BRAF^mut^ cells Hmel1^V600K/wt^ and M3^V600E/V600E^. The Chemio-Invasion assay was performed with at least three replicates per experimental condition. Scale bar: 200 μm. Magnification: 200×.

We subsequently exposed MM cells to 10 nM r-irisin, corresponding to the dose of irisin reported to exhibit biological activity in vitro [37], to assess whether irisin might interfere with cancer cell invasiveness across the extracellular matrix (ECM). As shown in Figure 2, MM cells exposed to irisin, used as a chemoattractant, invaded the ECM differently. Only LND1^wt/wt^ cells showed a significant reduction in invasion ability compared to cells not exposed to irisin (*p* < 0.05), while HBL, Hmel1 and M3 cells showed no change in invasiveness when compared to their corresponding untreated cells.

### 2.3. Alpha-V Integrin Expression in Metastatic Melanoma Cells

**Figure 3:**
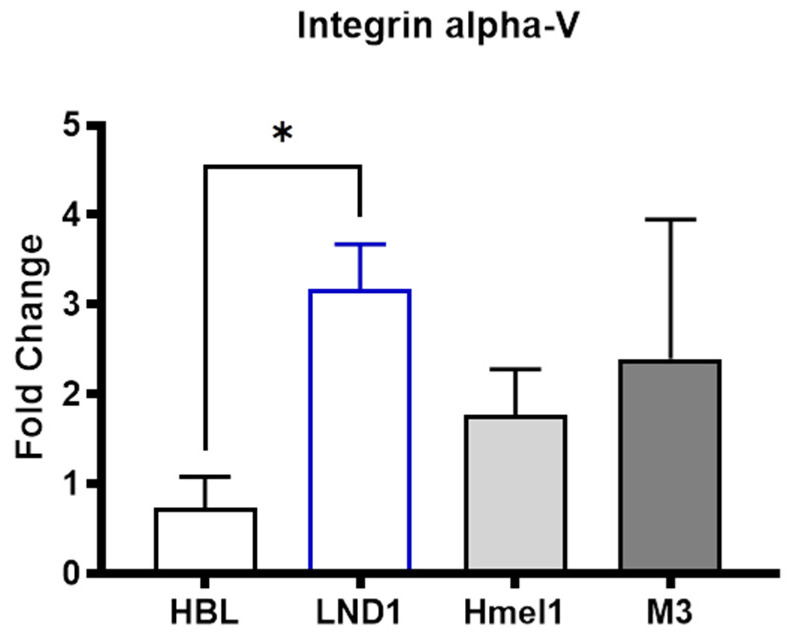
qRT-PCR analysis of alpha-V integrin. Alpha-V gene expression in different MM cell lines. Experiments were performed in triplicates. The histograms represent the means while the error bars present ±SD. * *p* < 0.05.

As integrin αV/β5 has been reported as the receptor for irisin on osteocytes, we measured the basal gene expression levels of integrins in the different metastatic melanoma cell lines. The gene levels of alpha-V integrin were differentially expressed between the two wild-type metastatic melanoma cell lines (Figure 3), while no differences where observed for beta-5 subunit.

As reported in Figure 3, *alpha-V integrin* is expressed at significantly higher levels only in LND1 cells compared to HBL, while its levels are not statistically different in Hmel1 and M3 mutated cells or in MM^wt^ cells, suggesting that the different expression of alpha-V integrin might be involved in the modulation of LND1 cell invasion.

### 2.4. Irisin Impairs the Expression of uPA/uPAR System

**Figure 4:**
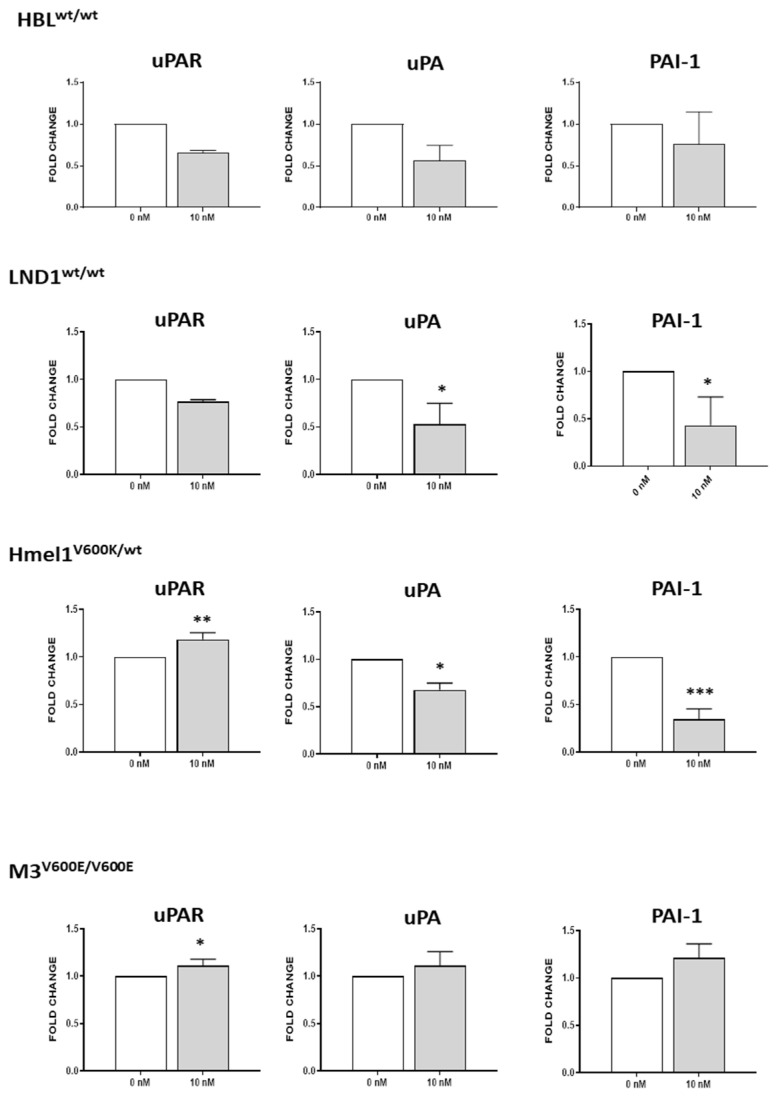
qRT-PCR analysis. Gene expression of the urokinase plasminogen activator receptor (uPAR), urokinase plasminogen activator (uPA) and its inhibitor PAI-1 in the different MM cell lines. Experiments were performed in triplicates. Histograms represent means while error bars represent ±SD. Unpaired two-tailed *t*-test. * *p* < 0.05; ** *p* < 0.01; *** *p* < 0.001.

We therefore studied whether irisin was able to influence the expression of the pro-invasive systems. Metastatic melanoma progression is mediated by the activation of the urokinase plasminogen activator receptor (uPAR), the urokinase plasminogen activator (uPA) and its inhibitor PAI-1, as well as the gelatinase system, including the metalloproteinases MMP-2 and MMP-9 and their inhibitors TIMP-1 and TIMP-2 [32]. We treated melanoma cell lines with 10 nM r-irisin to determine the level of the mRNA expression of ECM remodeling factors. As shown in Figure 4, HBL showed a trend of downregulation of uPA and *uPAR*, while LND1 showed a significant reduction in *uPA* and *PAI-1* (*p* < 0.05) upon irisin treatment compared to untreated cells, respectively. On the other hand, the BRAF^mut^ cells Hmel1^V600K/wt^ and M3^V600E/V600E^ exhibited a clear up-regulation of *uPAR* following irisin treatment compared to untreated cells (*p* < 0.05). In contrast, there was a decrease in the expression of *uPA* and *PAI-1* in the Hmel1^V600K/wt^ cell line, while no variations were observed in these genes in the M3^V600E/V600E^ cell line.

**Figure 5:**
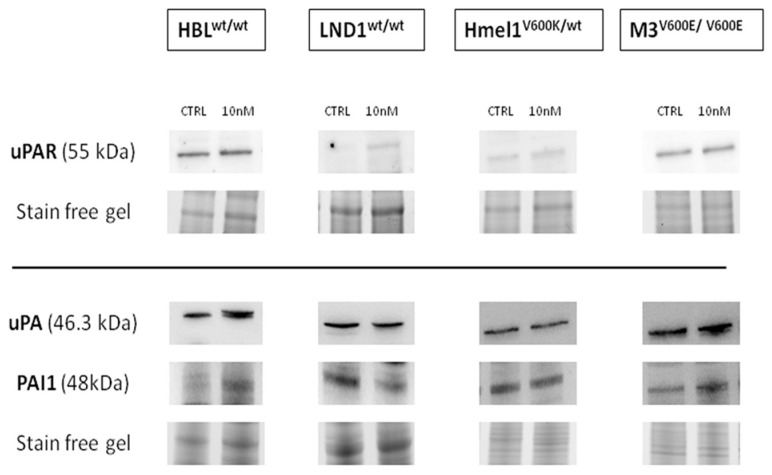
Western blot analysis. Representative immunoblots showing uPAR, uPA, and PAI-1 protein expression in the MM cell line treated with r-irisin 10 nM. Stain-free staining of total proteins loaded onto the gel was carried out for normalization by densitometric analyses, as described in Section 4.

As shown in Figure 5, the bands corresponding to proteins within the fibrinolytic system, namely uPAR, uPA, and PAI-1, exhibit trends consistent with qRT-PCR. Irisin treatment led to a decrease in uPAR protein expression in BRAF^wt^ cells, with reductions of 12.10% and 30.27% in HBL and LND1, respectively. Conversely, in BRAF^mut^ cells, irisin treatment resulted in an increase, with a rise of 4.67% in M3 and 37.53% in HMEL-1. Likewise, uPA expression decreased significantly following treatment, with reductions of 42.36% and 7.11% in HBL and LND1, and decreases of 10.90% and 46.46% in HMEL-1 and M3, respectively. Regarding PAI-1, irisin induced a 7.05% increase in HBL, a 28.55% reduction in LND1, and a 4.67% reduction in M3. Notably, in HMEL-1, PAI-1 protein levels increased by 37.53% after irisin treatment. In HMEL-1 cells, the observed increase in uPAR might imply a notable enhancement in invasive capabilities. However, this enhancement is presumably countered by the concurrent increase in PAI-1 levels and the reduction in uPA expression.

### 2.5. Irisin Modulates the Expression of the Gelatinase System

**Figure 6:**
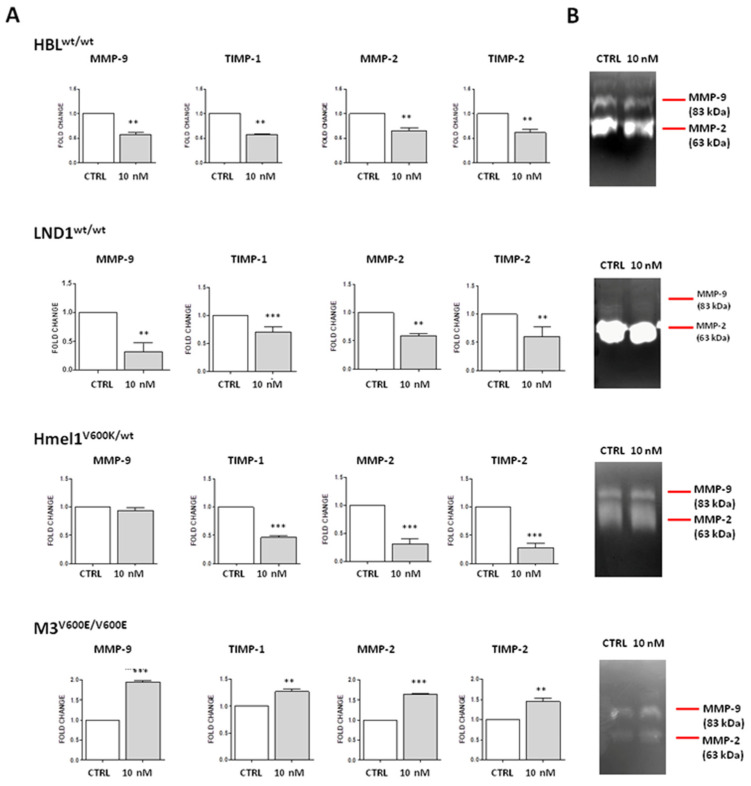
**Analysis of gelatinase system.** (**A**) Gene expression pattern of the gelatinase system (MMP-9, MMP-2, TIMP-1 and TIMP-2) in the four different MM cell lines following r-irisin treatment. Experiments were performed in triplicates. Unpaired two-tailed *t*-test. *p* < 0.01, **; *p* < 0.001 ***. (**B**) Zymography assay for MMP-9 and MMP-2 in the four different MM cell lines following irisin treatment.

Considering the gelatinase system, as shown in Figure 6A, irisin treatment significantly downregulated the expression of both MMPs and their inhibitors in wt MM cell lines compared to untreated cells (*p* < 0.001 ***). We also noticed that with regard to the BRAF^mut^ cell lines, the Hmel-1 cell line, which is heterozygous for the mutation in BRAF ^V600K/wt^, followed the same general pattern (*p* < 0.01 **) as the wt cells, except for *MMP-9*, the expression of which was unchanged following treatment compared to that of untreated cells. On the contrary, the M3 cell line, which carries a homozygous mutation in BRAF^V600E/V600E^, displayed a different scenario; indeed, irisin treatment increased the expression of *MMP-2* and *MMP-9* and their inhibitors, *TIMP-2* and *TIMP-1*, respectively (*p* < 0.001 ***).

These data were also supported by the gelatine zymography assay. As shown in Figure 6B, BRAF^wt/wt^ HBL and LND1 cell lines showed a reduction in the activation of both irisin-related MMP-2 and MMP-9. Regarding the BRAF^mut^ cell line, while the heterozygous Hmel1^V600K/wt^ showed no change in enzymatic activation state following irisin treatment compared to the untreated cells, the homozygous M3^V600E/V600E^ showed gelatinase activation following irisin treatment compared to the untreated cells.

## 3. Discussion

In 2012, Bostrom et al. showed that irisin, a segment of FNDC5 released from skeletal muscle, represents a link between exercise and metabolic homeostasis, enhancing the browning of white adipocytes in mice, leading to an increase in total body energy expenditure, a reduction in body weight, and an increase in insulin sensitivity [6,38]. Irisin is also associated with bone mineral density and strength in athletes and can enhance the differentiation of bone marrow stromal cells into mature osteoblasts [39,40]. Accordingly, irisin has profound effects in enhancing mass and improving the geometry and strength of cortical bone, specifically. Irisin may therefore not only be the molecule responsible for muscle–bone connectivity, but could also become a therapy for sarcopenia and osteoporosis, which occur in tandem in the elderly [41].

In addition to its metabolic effects, more and more findings revealed that irisin may also have a role in other systems, including cancer [20]. Indeed, irisin reduced the proliferation and migration capacity of breast [12], lung [13], prostate [14], osteosarcoma [15], pancreatic [16,17] and glioblastoma [18] cancer cells. However, the biological role played by irisin on melanoma cells has not been explored. In this context, elucidating the biological effect of irisin also in melanoma cells could improve the melanoma cell response to treatments, therefore reducing melanoma progression.

Cells adhere to ECM primarily by using vitronectin receptors of the integrin type, particularly αV/β3 and αV/β5 [42,43]. Acting as a molecular link between cells and their milieu (i.e., the ECM), integrins boost cellular dynamic processes such as adhesion, migration, and extravasation [44,45]. In normal epithelial cells, the expression of integrin αV/β3 is low, whereas high levels have been reported in melanoma [46]. Expression of the integrin αV/β3 has also been linked to the malignant progression of melanoma cells [47]. Analysis of a melanoma biopsy showed that integrin αV/β3 is a specific marker of the most malignant cells, suggesting a role for this adhesion receptor in the malignant growth of human melanoma tumors [48,49]. Intriguingly, irisin has been shown to bind to integrin αV/β5 in osteocytes [2,4]. Furthermore, αV/β5 integrin is also involved in the highly aggressive phenotype of melanoma cells such as cilengitide, an arginine–glycine–aspartic acid (RGD) broad-spectrum integrin inhibitor peptide [50], as well as reduced ECM invasion and the secretion of and MMP-9 by melanoma cells [51].

In our study, in order to unveil the biological effect of irisin on MM cells, we treated MM cell lines with different concentrations of irisin for 24 h and 48 h. We did not observe significant differences in the proliferation of MM cells during treatment compared to that of untreated cells. Interestingly, irisin was able to reduce the invasiveness of LND1^wt/wt^ melanoma cells, which highly express the αV integrin subunit at gene levels, suggesting its possible involvement in the irisin-mediated effect. Differently, the myokine did not impair the invasiveness of HBL^wt/wt^ or BRAF^mut^ cell lines. Noteworthy is the effect of irisin on the uPA/uPAR system and gelatinase system. In particular, we observed a significant reduction in their levels in BRAF^wt/wt^ cell lines (HBL and LND1) and BRAF^V600K/wt^ (Hmel1), while a slight increase was observed in BRAF^V600E/V600E^ (M3). Moreover, the decrease in protein levels, shown by Western blot analysis, in the BRAF^wt/wt^ cell lines were consistent with the outcomes of the fibrinolytic system’s gene expression. Accordingly, MMP-2 and MMP-9, as well as their inhibitors, showed a significant decrease both at the gene and the protein levels in BRAF^wt/wt^ and BRAF^V600K/wt^, while an opposite trend was observed in BRAF^V600E/V600E^.

Collectively, our results suggest that the fibrinolytic system and MMP-2/MMP-9 gelatinases were down-regulated in BRAF wild-type melanoma cell lines, while differential expression was observed in BRAF^mut^ cell lines, highlighting the complexity of the response to irisin in MM cells with different metastatic potential. Interestingly, the high expression of alpha-V integrin in LND1 cells and their response to irisin as a chemoattractant might suggest a possible role for this signaling pathway in the invasiveness of these cells. However, that irisin treatment also affects the uPA/uPAR system and gelatinases, independently from the different αV expression levels in different melanoma lines, suggests that not only might the integrin receptor be involved, but so might other signaling pathways and intracellular mechanisms, and this should be further investigated.

Considering the complexity of the response to irisin in different genetic backgrounds, it is possible that irisin modulates upstream components of these pathways or activates alternative signaling pathways that can counterbalance the effects of BRAF mutations. Indeed, irisin appears to exert its effects through multiple mechanisms and may interact with various types of receptors rather than a single, specific receptor, inducing pleiotropic effects on various tissues and organs [52]. The use of a CRISPR/Cas9 knockout screen targeting genes upstream of BRAF or involved in related signaling pathways could help identify genes whose loss affects the cellular response to irisin. Furthermore, as irisin is a soluble factor and integrins are anchored to the cell membrane, crystallography techniques would be required to investigate direct integrin–irisin interaction in melanoma cells. Preclinical studies using xenograft models with BRAF wild-type and mutant cell lines would also be beneficial to assess irisin’s in vivo therapeutic potential, including its efficacy, potential synergistic effects with existing therapies and impact on drug resistance.

Further research is warranted to fully elucidate irisin’s mechanisms of action in melanoma too, and to explore its potential as a therapeutic agent.

## 4. Materials and Methods

### 4.1. Cell Culture and Irisin Treatment

In the current study, four metastatic melanoma (MM) cell lines were utilized, HBL and LND-1 BRAF wild-type cell lines and BRAF-mutated (BRAFV600) Hmel-1 and M3. Melanoma cells HBL and LND1 gifted from Prof. G. Ghanem, Université de Bruxelles, Belgium. Hmel-1 and M3 were extracted from skin metastases obtained from human sporadic melanoma biopsy specimens after the informed consent of patients was provided. All cell lines were genotyped for BRAF as reported in Table 1, as previously described [25,53,54]. Cells were grown in high-glucose Dulbecco’s modified Eagle’s medium (DMEM) supplemented with 2 mM glutamine (Euroclone S.p.a., Pero, Italy), 100 UI/mL penicillin, and 100 μg/mL streptomycin at 37 °C in a humidified atmosphere with 5% CO_2_. All materials for cell culturing were purchased from EuroClone, Italy (Euroclone S.p.a., Pero, Italy). Human recombinant irisin (r-irisin) [AdipoGenLife Sciences, Liestal, Switzerland] was dissolved in dH_2_O and diluted with DMEM at the specific concentrations for the treatments.

### 4.2. MTT-Assay

MM cells were seeded in 96-well culture plates at a density of 5000 cells/well, and after 24 and 48 h, the culture medium was replaced with fresh medium (100 μL), including r-irisin at concentrations of 0 nM, 10 nM, 20 nM, 50 nM, 100 nM. The cells were treated for 24 h and 48 h, and cell viability was assessed by an MTT assay. Results are expressed as % cell viability at the tested doses and are reported as dose–response curves, obtained using CalcuSyn v.1.1.1 software (Biosoft; Acropolis Computers Ltd., Cambridge, UK).

### 4.3. Chemio-Invasion Assay

The invasion ability of MM cells was evaluated either in the absence or presence of irisin, at a concentration of 10 nM, as previously described [55]. Briefly, 8 μm pore size polycarbonate filters (Neuro Probe, Inc., Gaithersburg, MD, USA) were coated with Matrigel (Corning, Inc., Corning, NY, USA) and placed in Boyden chambers. Then, 8 × 10^3^ cells were plated onto the upper side of the chamber, while irisin was dissolved in the lower compartment of the chamber. The invasion was allowed to occur for 6 h for HBL and M3 and 18 h for LND1 and Hmel1. After incubation at 37 °C, the filters were fixed by methanol and the cells on the upper surface were removed by a cotton swab. The cells that migrated onto the lower side of the filter were stained by Diff-Quick (Mertz-Dade AG, Dade International, Milan, Italy) and counted using a light microscope (OLYMPUS CKX41, Tokyo, Japan) on the whole migration surface per well. Experiments were performed in triplicate. Invasion was expressed as the number of invasive cells ± SD of the number of total cells counted/filter.

### 4.4. Quantitative Reverse Transcription–Polymerase Chain Reaction (RT-PCR) Analysis

The 2 × 10^6^ MM cells were treated with r-irisin (10 nM) for 6 h and 18 h depending on the different cell types. The mRNA expression of uPA, uPAR, PAI-1, MMP-2, MMP-9, TIMP-1 and TIMP-2 was assayed from 1 μg of total RNA using the SYBR Green assay, as described previously [56]. The relative quantity of the gene expression was measured using the Applied Biosystems StepOne Real-Time PCR System (Applied Biosystems, WLM, Woburn, MA, USA). The results of three independent experiments run in triplicate were expressed as fold changes according to the 2^−ΔΔCT^ method [57] using 18S as a housekeeping gene, while for αV, integrin gene expression was normalized to β-actin. The sequences of specific primers are reported in Table 2.

### 4.5. Gelatin Zymography

MM cell lines were growth in the presence or absence of r-irisin (10 nM) for 6 h and 18 h. The conditioned medium was collected, and a gelatine zymogram was performed in order to evaluate the gelatinase activity. Aliquots of the culture medium (40 μg protein) were subjected to SDS-PAGE (8%, containing 0.13% gelatin) under non-reducing conditions. The gels were washed twice in a 2% Triton X-100 solution to remove the excess of SDS from the running gel and then incubated overnight at 37 °C in the presence of an incubation buffer. The day after, the gels were stained with a saturated Coomassie blue solution and then detained by several rounds of washing as previously described [56]. The enzymatic activity on the gelatin substrate was visible through transparent bands and was evident in the otherwise homogeneous blue gel.

### 4.6. Western Blot

Melanoma cell lines were grown to 70% confluence and treated with r-irisin at a concentration of 10 nM. An amount of 60 μg of cell protein lysate was run on 4–20% Mini-PROTEAN^®^ TGX Stain-Free™ Gel (Bio-Rad Laboratories, Inc., Hercules, CA, USA). The protein was transferred to a nitrocellulose membrane (Trans-Blot^®^ Turbo™ Mini Nitrocellulose, Bio-Rad Laboratories, Inc., Hercules, CA, USA) using the Trans-Blot^®^ Turbo™ Transfer System (Bio-Rad Laboratories, Inc., Hercules, CA, USA). The membrane was blocked and then incubated overnight at 4 °C with the following primary antibodies: uPA (CAT. N. TA805243, Origene Technologies, Rockville, MD, USA), PAI-1 (CAT. N. MAB-17171, Invitrogen, ThermoFisher Scientific, Waltham, MA, USA) and uPAR (MON-R-4-02, Invitrogen, ThermoFisher Scientific, Waltham, MA, USA) at the dilutions indicated by the manufacturer. In particular the primary antibody to uPAR (MON-R-4-02, Invitrogen, ThermoFisher Scientific, Waltham, MA, USA) was used in a non-reducing-condition according to the instructions provided by the manufacturer. On the following day, the membranes were incubated with the following secondary antibody: Goat Anti-Mouse IgG (H + L)-HRP Conjugate (dilution 1:3000, CAT. N. 1706516, Bio-Rad Laboratories S.r.l., Segrate, Italy). Blot detection was performed with ChemiDoc™ Imaging Systems (Bio-Rad Laboratories, Inc., Hercules, CA, USA). The Bio-Rad Image Lab 5.0 software was used for band intensity detection and quantification. The band intensity of each protein was normalized to the total protein amount loaded onto the gel.

### 4.7. Statistical Analysis

Statistical analysis was carried out using GraphPad Prism v8.0 (GraphPad Software, Inc., San Diego, CA, USA) software. Student’s *t*-test was applied to test significance. Data were considered statistically significant when *p*-value ≤ 0.05.

## Figures and Tables

**Table 1:** BRAF genotype analysis of melanoma cells lines used in this study.

Cell Line	Origin	BRAF Exon 15
HBL	Metastasis	WT/WT
LND1	Metastasis	WT/WT
Hmel1	Metastasis	V600K/WT
M3	Metastasis	V600E/V600E

**Table 2:** List of primers used in this study.

Target	Primer Sequence	5′ → 3′
*uPA*	forward	AGTGTCAGCAGCCCCACT
	reverse	CCCCCTGAGTCTCCCTGG
*uPAR*	forward	GCCCAATCCTGGAGCTTGA
	reverse	TCCCCTTGCAGCTGTAACACT
*PAI1*	forward	CTCCTGGTTCTGCCCAAGT
	reverse	GAGAGGCTCTTGGTCTGAAAG
*MMP-2*	forward	AGCACCGCGACAAGAAGTAT
	reverse	ATTTGTTGCCCAGGAAAGTG
*MMP-9*	forward	GACAAGCTCTTCGGCTTCTG
	reverse	TCGCTGGTACAGGTCGAGTA
*TIMP-1*	forward	GGGACACCAGAACTCAACCA
	reverse	GGCTTGGAACCCTTTATACATC
*TIMP-2*	forward	AAGCGGTCAGTGAGAAGGAA
	reverse	TCTCAGGCCCTTTGAACATC
*αV*	forward	AATCTTCCAATTGAGGATATCAC
	reverse	AAAACAGCCAGTAGCAACAAT
*18S*	forward	CGGCTACCACATCCAAGGAA
	reverse	GCTGGAATTACCGCGGCT
*β-actin*	forward	GCCGCCAGCTCACCAT
	reverse	AATCCTTCTGACCCATGCCC

## Data Availability

Data is contained within the article.
